# Spatial Characterization of Urban Vitality and the Association With Various Street Network Metrics From the Multi-Scalar Perspective

**DOI:** 10.3389/fpubh.2021.677910

**Published:** 2021-06-07

**Authors:** Chuanglin Fang, Sanwei He, Lei Wang

**Affiliations:** ^1^Institute of Geographic Sciences and Natural Resources Research, Chinese Academy of Sciences, Beijing, China; ^2^School of Public Administration, Zhongnan University of Economics and Law, Wuhan, China; ^3^Key Laboratory of Watershed Geographic Sciences, Nanjing Institute of Geography and Limnology, Chinese Academy of Sciences, Nanjing, China; ^4^Department of Planning and Environmental Management, University of Manchester, Manchester, United Kingdom

**Keywords:** urban vitality, spatial design network analysis, spatial scales, big data, Wuhan

## Abstract

In the context of rapid urbanization in developing countries, the spatial organization of cities has been progressively restructured over the past decades. However, little has been done to understand how the physical expansion affected the reorganization of socioeconomic spaces in cities. This study explores the association between various street network metrics and urban vitality and how it changes across different scales using geographic big data through a case study of Wuhan, China. Urban vitality is characterized by four components: concentration, accessibility, livability, and diversity. The new technique of spatial design network analysis (sDNA) is employed to characterize street network metrics, including connectivity, closeness, betweenness, severance, and efficiency, with 16 localized network variables. Furthermore, the stratified spatial heterogeneity between street network metrics at multiple scales and the four components of urban vitality is investigated using the Geodetector tool. First, concentration, accessibility, and diversity decline with distance from the urban center, whereas livability has a fluctuating upward trend with distance from the urban core. Second, the correlation between street network characteristics and urban vitality is sensitive to different spatial scales. Third, connectivity explains the largest amount of the variance in urban vitality (over 40%), while both betweenness and closeness explain roughly 28% of urban vitality. Efficiency and severance contribute 22 and 10% to the spatial heterogeneity of urban vitality, respectively. The study sheds light on the mechanisms between street configurations and urban vitality from the multi-scalar perspective. Some implications are provided for the improvement of the streets' urban vitality.

## Introduction

The world is experiencing rapid urbanization, especially in developing countries (1). By 2050, two thirds of the world's population will reside in cities. Although urbanization visibly improves standards of living, it also carries risks, such as shrinking or ghost cities^1^, which are associated with considerably low urban vitality (2–5). As an important proxy for the sustainability of urban growth (6), urban vitality measures the attractiveness and competitiveness of a city. As a new source of urban competitiveness, urban vitality helps a city gain comparative advantages and thus produce sustainable economic growth and endure regional innovation (7, 8). Understanding urban vitality is essential to urban health monitoring, compact urban development, innovative urban growth, and people-oriented urbanization.

The concept of urban vitality revolves around people's satisfaction with all aspects of urban life (9). It is difficult to capture the extensive meanings of urban vitality in specific measures. Many scholars have tried to measure urban vitality from different aspects. Yue et al. (10) measured urban vitality by the dimensions of built environment, human activities, and human–environment interaction. Pugalis (11) subdivided urban space into economy, culture, and society, classifying urban vitality as economic, cultural, and social vitality. A quantitative model of the determinants of urban vitality can be an informative tool to achieve sustainability in spatial planning and urban design (12, 13). Following the conceptual framework proposed by Jacobs (14) and Gehl (15), many scholars define and quantify urban vitality in terms of multiple facets (e.g., density, accessibility, and diversity), multiple spatial scales (e.g., *jiedao* units, community neighborhoods, and street blocks), and multiple temporal horizons (e.g., night time, twilight, and early morning) (16, 17). The microscale analysis of communities or street blocks provides a granular and comprehensive perspective of the spatiotemporal dynamics of urban vitality in cities (18). Moreover, the advent of geographic big data such as location-based social media, points of interest (POIs), mobile Internet data, and other web crawler data allows researchers to capture spatial dynamics of urban vitality more accurately and at finer scales.

The association between urban form metrics and urban vitality has been widely recognized in the literature (19). Empirical studies of both developed and developing countries have examined the inherent vital nature in urban areas and provided different conceptualizations of urban vitality in various territorial contexts. Jacobs (14) proposed that land-use mixture, block size, age of buildings, density, accessibility, and border vacuums are the major components of measures of urban vitality in the United States. Using his framework, Delclòs-Alió and Miralles-Guasch (16) also interpreted urban vitality in Barcelona, Spain. Sung et al. (20) found an association between a diverse physical environment and walking activity on the streets in Seoul, South Korea. In addition, Long and Huang (21) found significant and positive influences of urban design variables such as land-use mix, road intersection density, and accessibility to various facilities on economic vitality for the 286 largest cities in China. Similarly, Yue et al. (10) discovered that urban vitality measurement is closely linked with built environment (e.g., buildings, blocks, and land types), human activities (e.g., concentration of residents, employees, and tourists), and human–environment interaction (e.g., infrastructure, road network, natural vacuums, and artificial segregations) in Shanghai, China. In the context of vitality debates worldwide and the prospects of future urban sustainability, it is emergent for modern planners to deeply analyze and realign the urban spatial structure to promote the necessary interactions and provide a sufficient physical environment for the socioeconomic dynamics. This study aims to better understand the spatial organization of cities from the multi-scalar perspective of street network design and how urban vitality is related to their distributions.

Previous studies have widely acknowledged that street configurations have significant effects on physical activities in streets and thus urban vitality (22). Focusing on the streets of Cypriot towns, Jalaladdini and Oktay (23) argued that good connections to the street, the harbor, or the historic quarter and proximity to important magnets are essential to understanding the issue of vitality in urban public spaces. Another study taking Dutch towns and new Chinese towns as examples reported that spatial configurations of a street network, in terms of topological, geometrical, and metric distances, directly determine economic vitality and encourage vibrant street life (24). Using network centrality indices, Kang (25) examined the effects of street network configurations on walking mobility in Seoul, Korea. While the existing literature has measured various aspects in terms of typology, geometrics, and network connectivity, the discussion has rarely considered the scale effect when measuring the street configurations. Notably, the metrics of the street layout can be quantified differently within the neighborhood environment as defined by various buffer widths (26). Hajrasouliha and Yin (27) argued that the gridiron street patterns with small or large blocks have various effects on pedestrian volumes, and the street networks with small block sizes help pedestrians to understand their surroundings better. He et al. (28) proved that the street configurations under walking or driving modes should be differently measured, which has an influence on the distribution of leisure entertainment facilities. Thus, it is critical to measure the street configurations from a multi-scale perspective and compare how urban vitality is associated with street configurations at different geographic scales.

In addition to the topological, geometric, and distance features of the street layout, the network metaphor has a long tradition in the analysis of urban planning and transportation (29, 30). More recently, the centrality assessment model and space syntax analysis have been used to evaluate the structural properties of street networks in an urban system. Street centrality indices representing closeness, betweenness, and straightness capture the skeleton of the urban system; these factors shape economic activities and land-use intensity (31, 32). Space syntax focuses on topological distance within a network and offers an effective tool to measure street connectivity (33). However, these techniques fail to capture the challenges of physical severance and network efficiency, especially the navigating difficulties and psychological barriers of pedestrians (28, 34). One of these measurements is directly mirrored in spatial design network analysis (sDNA), which incorporates six important features (density, connectivity, closeness, betweenness, severance, and efficiency) that are hypothesized to affect urban vitality in an urban system.

As a representative developing country, China has been undergoing rapid urbanization and economic growth. Driven by both industrialization and urbanization, numerous satellite towns, industrial zones, commercial centers, and residential areas have emerged at the urban fringe and pose significant challenges to sustainable urban development (35). Meanwhile, the opposite phenomenon of “shrinking cities” in China has been a catalyst for the proposal of the “National New-type Urbanization Plan (2014–2020),” which specifically stresses people-oriented urbanization and human well-being (36). Urban vitality was initially studied as an important path to new-type urbanization in North America. Until recently, empirical evidence in developing countries has indicated that the dynamic process and determinants of urban vitality can be different from the case studies in the United States or Europe due to their dissimilar urban morphology and spatial planning (37–39). Therefore, this study aims to enrich the existing empirical studies by taking an inland city of China as an example and formulating policy implications to enhance urban vitality and promote urban health.

This study contributes to the literature in the following three ways. First, due to the inconclusive evidence in the literature, this study aims to examine the inherent vital nature within a neighborhood community in an inland city of China using geographic big data. Second, as existing studies have rarely considered the scale effect when measuring street configurations, this study adopts a multi-scale perspective incorporating street networks to examine how urban vitality is associated with spatial network layouts at different geographic scales. Third, the study applies a newly developed technique called sDNA to capture the navigating difficulties and psychological barriers faced by pedestrians, which are hypothesized to affect urban vitality.

## Conceptual Framework

The association between street network configurations and urban vitality must be measured at multiple scales to assess the design-oriented characteristics of street networks that most influence street activities and then urban vitality. All networks are composed of nodes and links. Within street networks, nodes represent junctions or intersections between streets. As a specific type of spatial network, nodes always have distinct geographic locations, and links always have a physical shape (34). From the perspective of sDNA, the street network configurations—connectivity, closeness, betweenness, severance, and efficiency—as seen in Table 1, are hypothesized to affect urban vitality in an urban system.

**Table 1:** The description of street network configurations.

**Metric**	**Name (abbrev.)**	**Description**
Connectivity	Connectivity in radius (CONN)	The total number of link ends connected at each junction
	Junctions in radius (JNC)	The number of junctions in the radius
Closeness	Mean Euclidean distance in radius (MED)	The mean length between an origin and all destinations in the radius
	Network quantity penalized by distance in radius Euclidean (NQPDE)	The mean length of network weight is divided by network quantity in the radius
	Angular distance in radius (ANGD)	The total angular curvature on all links in the radius
Betweenness	Betweenness Euclidean (BTE)	The number of geodesic paths that pass through a vertex
	Two-phase betweenness Euclidean (TPBTE)	The sum of geodesics that pass through a link, weighted by the proportion of network quantity
	Two-phase Destination Euclidean (TPD)	The proportion of origin weight received by each destination in the two phase betweenness model
Severance	Mean crow flight distance in radius (MCF)	The mean of the crow flight distance between each origin and all links in the radius
	Diversion ratio in radius Euclidean (DIVE)	The mean ratio of geodesic length to crow flight distance over all links in the radius
	Mean geodesic length in radius Euclidean (MGLE)	The mean length in Euclidean metric of all geodesics in the radius
Efficiency	Convex hull area (HULLA)	The area of the convex hull covered by the network in the radius
	Convex hull perimeter (HULLP)	The perimeter of the convex hull covered by the network in the radius
	Convex hull maximum (crow flight) radius (HULLR)	The distance from the origin to the point where the convex hull has its greatest radius
	Convex hull bearing of maximum radius (HULLB)	The direction of the projected grid for HULLR
	Convex hull shape index (HULLSI)	The perimeter of the convex hull divided by the area of the convex hull

The connectivity aspect refers to the quantity of link ends connected at each junction and the number of junctions within the user-defined radius. More connected street networks tend to have many short links, numerous intersections, and minimal dead ends. Good street connectivity means providing various routes from residential neighborhoods to destinations such as schools and shops by walking or driving. The benefits of better connectivity include improved ease of mobility through the road network, high reachability from an origin to the desired destination, less traffic congestion, and a safer street environment (40). Street connectivity is significantly correlated with active transportation, which helps to create more walkable and livable communities (41). However, how street connectivity is associated with urban vitality remains to be studied.

The closeness aspect refers to the mean Euclidean distance, the network quantity penalized by distance, and the angular distance within the radius. As a form of network centrality, closeness measures the difficulty of navigating to all possible destinations from each link in the radii. This study emphasizes the significance of angular distance (distance measured in terms of angular change) and prefers to use the shortest angular paths instead of Euclidean distance. In reality, pedestrians and drivers tend to follow straight roads and angular geodesics because they are easier to remember and tend to be faster on average (42). The accessible locations for pedestrians and drivers are always linked with diversified land-use and a convenient street layout (43). The closeness measures can reflect accessibility and flow potential, which is conducive to creating a vibrant neighborhood.

The betweenness aspect measures “through-movement” on spatial networks. The conventional betweenness indicator is based on the idea that a vertex is central if it lies on the shortest path between other vertices. Previous studies have proven that betweenness largely determines the location of retail shops and services in the urban area (44). Higher betweenness is always associated with high housing prices and rent, traffic flow, population density, and the flow of commuting in street networks (45). This study proposes two new betweenness indices—two-phase betweenness and two-phase destination. The former calculates the betweenness weighted by the network quantity, while the latter measures that weighted by both the origin and destination. In a sense, the standard betweenness can be seen as an opportunity model, whereas the new betweenness indicators correspond to transport models with trip generation and distribution phases. The betweenness concept measures the complex flow of commuting and can produce vibrant economic activities.

The severance aspect belongs to network detour analysis and mainly measures the degree the network deviates each other from the most direct path. By comparing straight-line distance to actual network distance, the severance measures can effectively reflect the twistedness of the localized network (28). In this study, the severance indices include mean crow flight distance, diversion ratio, and mean geodesic length in the radius, which proxy the physical and psychological separation in the network. High severance represents the more cognitive difficulties of pedestrians or drivers when navigating the road network. The severance can reflect the network characteristics in more detail and may indicate unfavorable locations for vibrant commercial activities.

The efficiency aspect belongs to network shape analysis and refers to the form of the overall spatial footprint within the user-defined network. It measures the overall efficiency of the network in a sophisticated manner by considering the shape of links and the spatial structure of link connections (34). High efficiency represents high frequent navigation through the network on foot or by automobile. While the road design in the urban center does not provide an efficient walking environment for pedestrian interaction, the major roads connecting the urban core and urban fringe always have low efficiency for driving (28). An efficient network tends to create more opportunities for daily interaction such as relaxation and leisure activities.

The key component of measuring street network metrics is to choose a spatial scale of interest. This defines how much of the “surrounding network” we consider when computing statistics for each individual link. Corresponding to urban vitality, the spatial scale possibly refers to the surrounding environment within a specific network radius where most daily activities take place. The network radius can be defined by the distance from either walking or driving (34). The multi-scalar perspective compares how each street network metric is spatially associated with urban vitality over five different scales of interest (500, 1,000, 1,500, 2,000, and 2,500 m). There is no single optimal scale for assessing urban vitality and designing street networks. The existing literature has rarely compared how different urban forms are associated with social interaction, physical activities, and land-use diversity. Thus, the spatial explicit analysis between the multi-scalar street network metrics (e.g., density, connectivity, closeness, betweenness, severance, and efficiency) and urban vitality yields important information for human-scale street design and land-use planning.

## Methods

### Description of the Study Area

At a longitude of 11341′ ~ 11505′ and a latitude of 2958′ ~ 3122′, Wuhan is situated in the eastern part of Hubei and is the largest megacity in central China. In 2018, the permanent population of Wuhan amounted to 11.08 million people, and the gross domestic product (GDP) for the city was 1.48 trillion RMB. Per capita GDP in Wuhan (135,136 yuan) ranked first among cities in central China. Primary, secondary, and tertiary industries comprise 2.4, 43.0, and 54.6% of the economy, respectively, with the service industry being dominant. The multimodal street network in Wuhan, known as a national bus city in China, is highly developed and includes roadways, subways, railways, waterways, and greenways. The local government has emphasized transit-oriented development to promote smart urban growth, inject vitality into old towns, and create a more sustainable transport system.

The central city of Wuhan is divided by the Yangtze River and the Han River into seven districts. There are 89 *jiedaos* and over 1,076 community neighborhoods^2^ in central Wuhan. Most physical activities of human life take place in the public spaces of community neighborhoods. As the center of human activities, the community neighborhood provides an important space for people to live, rest, socialize, produce, and work. Thus, a fine spatial scale is necessary to capture the details of street configurations and the patterns of residents' social interaction in daily life.

There are several historic community neighborhoods in the inner city of Wuhan. As the old residential quarters, these neighborhoods face difficulties associated with aging municipal public infrastructure and a lack of public services. Since 2016, the local government has focused on urban redevelopment and advanced some reform policies to reconstruct these areas. Meanwhile, because of traffic congestion and poor living conditions in the urban core, more people have chosen to live in the suburbs, which feature spacious housing and better community environments. Revitalizing old towns and strengthening vitality in the suburbs are important issues for the local government. This study employs a spatial network analysis tool to characterize the distribution of urban vitality and investigate how street network designs can create a vital city.

### Data Sources

#### Demographic Data

The 6th National Census survey in 2010 provides precise demographic data aggregated to the administrative boundaries (provinces-prefectures-counties/urban districts-townships/*jiedao*s). As the most detailed demographic data can only be obtained by the public at the *jiedao* level, the demographic data at the scale of community neighborhood are missing. The WorldPop dataset provides gridded population maps with 100-m spatial resolution for each country in the dataset. However, Ye et al. (46) find that the WorldPop dataset underestimates the population in urban areas and overestimates it in rural areas of the Chinese mainland. Thus, this paper utilizes the improved population images developed by Ye et al. (46), with higher accuracy than WorldPop for China by integrating remotely sensed and POI data within a random forest model.

#### Points of Interest Data

POIs refer to all geographic entities that can be abstracted into points. Each POI observation contains information such as latitude, longitude, names, and addresses. The POI data in this study are taken from the Baidu map (http://map.baidu.com/). There are 66,161 POIs within the study area in 2016, including the following: shopping malls, petrol stations, restaurants, tourist attractions, banks, parks, chess and card rooms, theaters, karaokes, stadiums, bars, hospitals, hotels, bus stations, universities, ATMs, and government agencies. These POI data can be categorized into nine classes: financial service facilities, research and educational facilities, cultural facilities, health service facilities, leisure and recreational facilities, commuting facilities, government agencies, catering services, and lodging services. The POI data reflect the venues of social activities and identify the functional diversity in the urban core.

#### Additional Geographic Big Data

This study utilizes additional geographic information about housing prices and age to measure the livability of community neighborhoods. This information is collected from *Fangtianxia*, the largest real estate transaction platform in China (http://fang.com). The kriging interpolation method^3^ is applied to obtain the spatial pattern of housing prices and age with a spatial resolution of 100 m in Wuhan. Building density is an important indicator that reflects the concentration aspect of urban vitality. WorldView-2 image with a high spatial resolution of 0.6 m is utilized to draw the building bases in the study area. Subsequently, it is overlaid with the boundary of each community neighborhood to obtain the building density of each spatial unit. ArcGIS 10.2 is employed to calculate the Euclidean distance between each community neighborhood and all bus stops and subway stations to measure accessibility.

### Method

#### Quantification of Urban Vitality

Urban vitality is measured with four major components: concentration, accessibility, livability, and diversity. A dense concentration of people, buildings, and social activities is the most basic condition for ensuring that an urban area is vital. The concentration component can be evaluated by three variables: population density, building density, and POI density. According to Jacob (14), a vibrant city also requires high accessibility on foot and by public transport, contrary to car-dependent urban planning. The accessibility component can be quantified in terms of distances to bus stops and subway stations. If a certain balance between new and aged buildings is maintained, diversity from both a land-use and social perspective can be strengthened (47). A precarious housing market and high house prices are likely to cause residential segregation and social polarization. The livability component can be assessed by housing prices and age. Highly diversified neighborhoods and streets shape urban vitality mainly by increasing social interactions. The diversity component is evaluated by land-use diversity. The formulas for calculating concentration, accessibility, livability, and diversity are as follows:

(1) $$\begin{array}{l} Con_{i}=f\left(popd_{i},\;bd_{i},\;POID_{i}\right) \end{array}$$

(2) $$\begin{array}{l} Acc_{i}=f\left(dis\text{\_}bs_{i},dis\text{\_}sw_{i}\right) \end{array}$$

(3) $$\begin{array}{l} Liv_{i}=f\left(hage_{i},\;hpr_{i}\right) \end{array}$$

(4) $$\begin{array}{l} Div_{i}=f\left(landuse_{i}\right),\;landuse_{i}=\;-\overset{n}{\underset{i=1}{\sum }}\left(p_{i\;}\times 1n\;p_{i\;}\right) \end{array}$$

In the final equation, *p*_*i*_ is the proportion of the *i*^*th*^ POI type among the total number of POI records, and *n* is the total number of POI types. The weights of all variables are determined by the entropy method.

#### Spatial Stratified Heterogeneity Analysis

Spatial heterogeneity characterizes the local variance of spatial dependence (48). Spatial stratified heterogeneity, which stresses the between-strata variance more than the within-strata variance, is used in many fields of natural and social sciences. The geographic detector developed by Wang et al. (49) is a new statistical technique for detecting spatial differentiation and investigating the factors that drive this spatial phenomenon. This tool consists of four functions: factor, interaction, risk, and ecological detectors. This study primarily employs the factor detector to examine the spatial stratified heterogeneity of the dependent variable *Y* (urban vitality) and the determinant power of independent variables *X* (various street network metrics). The contribution of the covariate *X* to the spatial heterogeneity of urban vitality can be formulated by the *q* statistic as follows:

(5) $$\begin{array}{l} q=1-\frac{SSW}{SST} \end{array}$$

(6) $$\begin{array}{l} SSW=\overset{L}{\underset{h=i}{\sum }}N_{h}\sigma_{h}^{2} \end{array}$$

(7) $$\begin{array}{l} SST=N\sigma^{2} \end{array}$$

where *SSW* refers to the within sum of squares, and *SST* is the total sum of squares. *h* = 1, 2, …., *L* is the strata of covariate *X*; *N*_*h*_ and *N* are respectively the number of spatial units in strata *h* and the whole area; $\sigma_{h}^{2}$ and σ^2^ refer to the variance of *Y* in strata *h* and the whole area, respectively.

*q* ∈ [0, 1] indicates that *X* contributes 100 × *q%* to the pattern of *Y*. A large value of *q* indicates the large contribution of *X* to *Y*. If *q* is equal to 0, then no association of *X* and *Y* is shown. If *q* is equal to 1, the distribution of *Y* can be completely explained by *X*.

## Result Analyses

### Spatial Characterization of Urban Vitality

**Figure 1:**
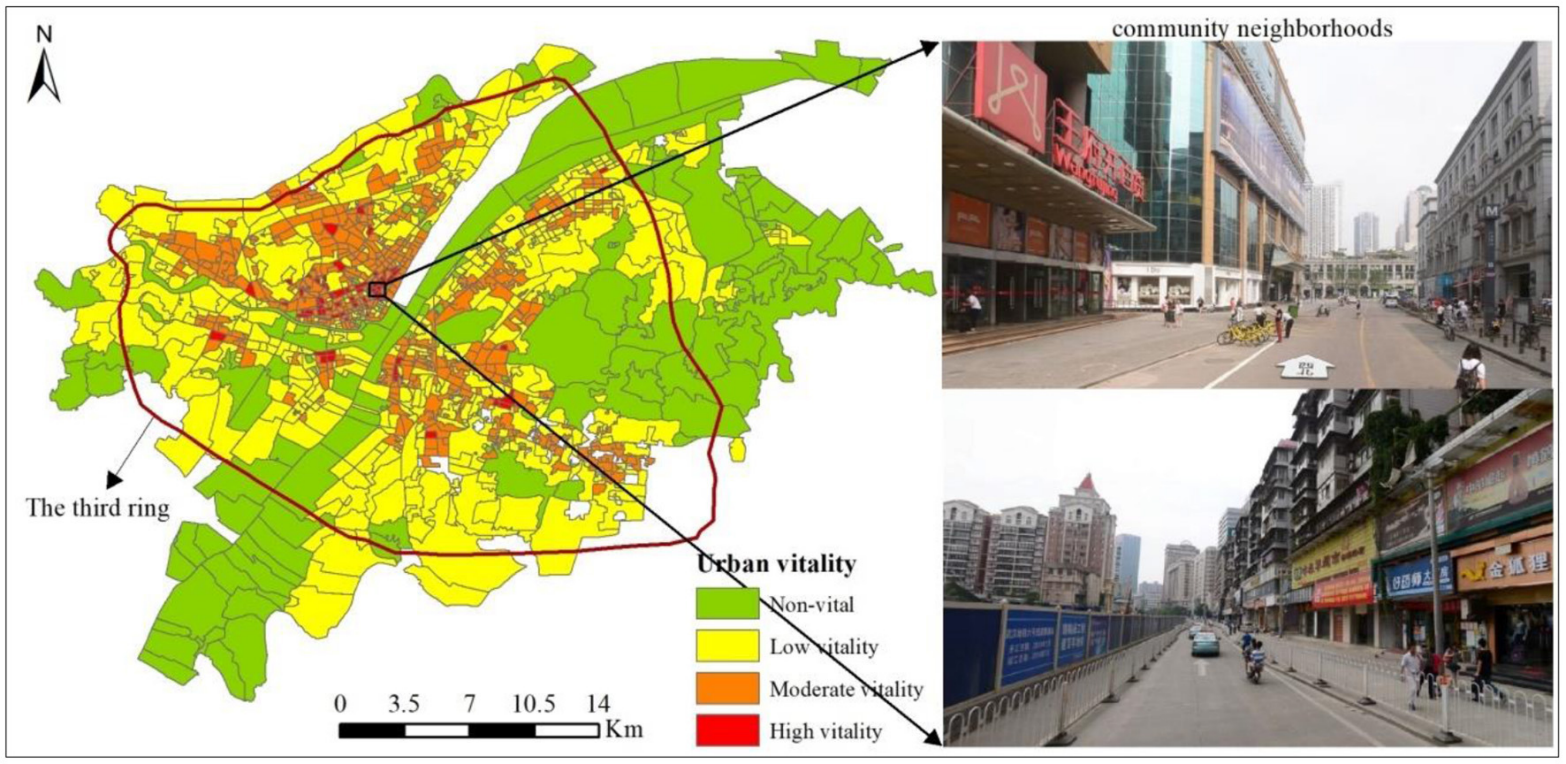
The spatial distribution of urban vitality and community neighborhoods with the highest value of urban vitality (Red denotes high vitality; orange denotes moderate vitality; yellow denotes low vitality; and green denotes non-vital areas).

**Figure 2:**
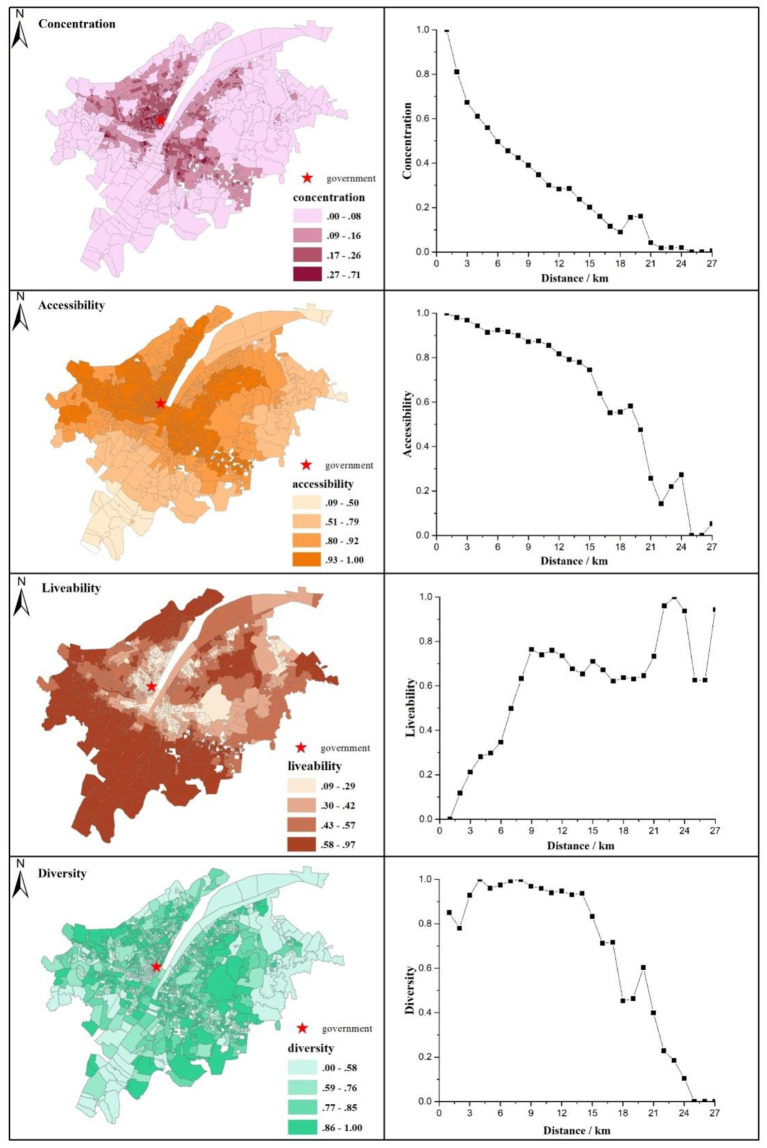
Spatial pattern of concentration, accessibility, livability, and diversity (the left column) along with concentration, accessibility, livability, and diversity in concentric rings with the distance to the city center (the right column) in Wuhan.

Figure 1 demonstrates the spatial distribution of urban vitality, showing the community neighborhoods with the highest value of urban vitality. The high values of urban vitality are mainly clustered in the downtown area, whereas the uptown area is characterized by low urban vitality. The community neighborhood with the highest value of urban vitality is located near the city government, namely, the Jianghan walking street, a famous century-old commercial street that features shopping, entertainment, tourism, and culture. In the grouping analysis, urban vitality is categorized into four classes in Figure 2: high vitality, moderate vitality, low vitality, and non-vital areas. High-vitality areas account for 7% of all community neighborhoods in Wuhan. These communities mainly correspond to the traditional city centers, which are characterized by dense population, pedestrian-friendly street networks, and diversified built environments. The moderate-vitality category takes up around 38% of all neighborhoods in the study area. These communities are regarded as the transition buffer between high- and low-vitality areas, and they are expected to have a certain level of vibrant street life. Respectively, 43 and 12% of community neighborhoods are categorized as low-vitality and non-vital areas. These areas are mainly located in the disadvantaged urban fringe near agrarian, natural, or industrial lands.

The left column in Figure 2 demonstrates the spatial patterns of concentration, accessibility, livability, and diversity. Overall, both concentration and accessibility decline as distance from the urban center increases, while livability and diversity show different patterns. A core-periphery pattern can be observed in urban concentration. The high values of concentration are mainly found in historical neighborhoods, which meet the requirements of Jacobs (14) of a dense distribution of people, buildings, and streets. Accessibility is primarily explained by a centric-periphery logic in which the downtown is equipped with good transport infrastructure. Areas near shopping malls, big city hospitals, municipal governments, office buildings, and schools have good accessibility. Conversely, while urban suburbs with low housing prices and good residential environments show good livability, the urban center has the majority of old residential buildings without elevators, sports facilities, or sufficient open spaces. However, as some successful urban renewal projects have recently been implemented in the downtown, livability within the urban core is expected to improve in the future. It is rather difficult to depict the pattern of diversity clearly. The neighborhoods near the Yangtze River and the Han River are less diversified as they are constrained by urban planning and aquatic environment protection.

The right column in Figure 2 depicts how concentration, accessibility, livability, and diversity change with the distance to the city center. As the key component of urban vitality in Jacobs' arguments, concentration decreases rapidly as the distance to the city center increases, despite an upturn in the interval between 18 and 21 km, where some new towns labeled “high-tech zones” or “ecological cities” are built. Characterized by convenient transportation, pleasant living environments, and more employment opportunities, these new towns are becoming the new urban center and attracting several people to live and work there. Accessibility slowly declines when the distance from the city center is <15 km. Subsequently, accessibility drops rapidly despite slight increases in remote urban new towns. However, livability increases sharply when the distance to the city center increases to 9 km, indicating the low livability in the downtown. Livability fluctuates slightly in areas between the second and third ring roads, increases sharply in the urban suburbs, and subsequently decreases rapidly. It seems that the urban fringe is rather uniformly unlivable, indicating the heterogeneous space of livability in the urban fringe. Diversity demonstrates an upward trend within the buffer of 4.5 km and subsequently levels off within the third ring road. Diversity decreases dramatically after the distance of 15 km despite a slight upturn in the new town.

### The Correlation Between Street Network Metrics and Urban Vitality

Spatial network analysis is conducted to create statistics that describe the multi-scalar configuration of street networks. Contrary to techniques such as street centrality, space syntax, and accessibility analyses, sDNA comprehensively describes network features, including centrality, network shapes, and the navigability of areas, at user-defined network scales. A key component of sDNA is the standardization of network links, which avoids the modifiable areal unit problem (MAUP) by dividing the network into individual links. Another key component of sDNA is the specification of a scale of interest. To detect the influence of different scales on urban vitality, this study chooses five spatial scales–500, 1,000, 1,500, 2,000, and 2,500 m—providing a wide range from sensible walking distances to driving distances.

A total of 16 variables representing density, connectivity, closeness, betweenness, severance, and efficiency are computed using the sDNA software developed by Cardiff University (https://www.cardiff.ac.uk/). These 16 localized network measures, shown in Table 2, are assumed to affect urban vitality in Wuhan. The Pearson correlations between these network variables and the neighborhood urban vitality are calculated at each of the five spatial scales to determine how these network characteristics are associated with urban vitality and at which scale the association is most evident.

**Table 2:** The correlation between street network metrics and urban vitality.

**Metric**	**Name (abbrev.)**	**Best correlation and radius**	**Metric**	**Name (abbrev.)**	**Best correlation and radius**
Connectivity	CONN	+0.65, 1,500 m	Severance	MCF	+0.23, 2,500 m
	JNC	+0.65, 1,500 m		DIVE	−0.34, 2,500 m
Closeness	MED	+0.28, 500 m		MGLE	+0.29, 500 m
	NQPDE	+0.66, 2,500 m	Efficiency	HULLA	+0.61, 500 m
	ANGD	+0.51, 2,500 m		HULLP	+0.53, 1,000 m
Betweenness	BTE	+0.60, 1,500 m		HULLR	+0.26, 2,500 m
	TPBTE	+0.53, 1,000 m		HULLB	+0.05, 1,000 m
	TPD	+0.29, 1,500 m		HULLSI	+0.07, 1,500 m

Density captures certain built environment features, such as the density of jobs and homes. The hypotheses behind these two variables are an optimum built environment density for urban vitality. The best correlation coefficients between dense built environment and urban vitality amount to 0.68 for LINK and 0.69 for LEN, both at the spatial scale of 1,000 m. The scale of 1,000 m implies the built environment within the walking distance. More density of street links is highly linked with higher urban vitality, possibly denoting that people prefer to walk within the 1,000-m neighborhood and thus produce more socioeconomic activities when navigating the street networks.

Connectivity measures how well streets are linked with others and the density of intersections. Neighborhoods with high street connectivity tend to have streets with several short links, numerous intersections, and few dead ends, which facilitate physical activities such as walking and cycling. The best correlation coefficients between connectivity variables and urban vitality reach 0.65 at the spatial scale of 1,500 m, which corresponds to the cycling distance. The best correlation between connectivity and urban vitality at the scale of 1,500 m represents that well-connected streets facilitate cycling within the neighborhood and then encourage more physical activities.

Closeness reflects the level of accessibility and reachability between origins and destinations. Previous studies emphasize the shortest Euclidean path to measure closeness; however, sDNA utilizes angular analysis to reflect the cognitive difficulty of navigation. The shortest angular path can reflect the subtleties in the network layout. According to the best correlation coefficients, network quantity penalized by distance in radius Euclidean (NQPDE) and angular distance in radius (ANGD) are more related with urban vitality at the spatial scale of 2,500 m than at other scales. The scale of 2,500 m corresponds to the driving distance. The high correlation between closeness and urban vitality at the scale of 2,500 m signifies that a street network design with less angular distance is conducive to driving and can encourage more diversified activities in the neighborhood.

Betweenness measures how street networks are populated with entities when traveling from origins to destinations in the user-specified radius. It involves all possible trips that pass through the link in the radius and can effectively reflect the flow volume through walking or traffic to the destination. Contrary to the normal betweenness model, the two-phase betweenness model considers a fixed amount of weight in each origin or destination according to the quantity of visits per link. The best correlation coefficients between these three variables and urban vitality are 0.60 for betweenness Euclidean (BTE) in the radius of 1,500 m, 0.53 for two-phase betweenness Euclidean (TPBTE) in the radius of 1,000 m, and 0.29 for two-phase destination Euclidean (TPD) in the radius of 1,500 m. The highest correlation between betweenness metrics and urban vitality at the scale of 1,000 or 1,500 m demonstrates that the street design facilitating walking or cycling can encourage mixed land-use and social interaction, which help to create a vibrant neighborhood.

Severance measures the opposite metrics of connectivity in network detour analysis and primarily reflects how the street network deviates from the most direct path, which proxies the navigating difficulties of pedestrians or vehicles by measuring the extent to which the local network is twisted. Diversion ratio in radius Euclidean (DIVE) is negatively correlated with urban vitality, and the best correlation coefficient is −0.34 at the spatial scale of 2,500 m. The negative correlation between street severance and urban vitality at the scale of 2,500 m represents that street network detours increase navigating difficulties of vehicles and are not conducive to vibrant commercial activities.

Efficiency measures the ease of navigation in network shape analysis considering the shape of links, the arrangement of links, and the number of connections. Convex hull area (HULLA) and convex hull perimeter (HULLP) are more associated with urban vitality than other variables, and the best correlation coefficient is 0.61 for HULLA at the spatial scale of 500 m and 0.53 for HULLP at the spatial scale of 1,000 m. The best association between efficiency metrics and urban vitality at the scale of 500 or 1,000 m denotes that the walking-friendly street shape is important for pedestrian interactions and creating a vital environment.

### Spatial Stratified Heterogeneity Between the Multi-Scalar Network Metrics and Urban Vitality

Table 3 demonstrates the *q* statistics between urban vitality and spatial network metrics at multiple scales. Overall, the density metric has the largest explanatory power (~46%) for the distribution of urban vitality at all scales. Dense street networks can increase people's contact opportunities, promoting economic and social activities. In the Chinese context, the downtown area is usually characterized by narrow streets and densely distributed networks, leading to high urban vitality. The two density metrics—LINK and LEN—have explained 46% of urban vitality at all spatial scales according to Table 2. Over increasing distances, the influences of both LINK and LEN on urban vitality increase, peak at the spatial scale of 1,000 m, and then monotonically decrease.

**Table 3:** *q* statistics between urban vitality and spatial network metrics at multiple scales.

**Metrics**	**Variables**	**500 m**	**1,000 m**	**1,500 m**	**2,000 m**	**2,500 m**	**Trend**	**Mean**
Connectivity	CONN	0.428	0.465	0.467	0.447	0.420	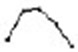	0.445
	JNC	0.427	0.465	0.469	0.444	0.415	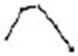	0.444
Closeness	MED	0.148	0.110	0.098	0.074	0.117	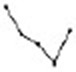	0.109
	NQPDE	0.329	0.435	0.468	0.459	0.471	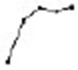	0.432
	ANGD	0.122	0.259	0.312	0.329	0.337	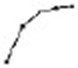	0.272
Betweenness	BTE	0.382	0.453	0.467	0.434	0.428	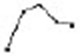	0.433
	TPBTE	0.307	0.317	0.322	0.269	0.288	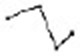	0.301
	TPD	0.057	0.089	0.109	0.109	0.111	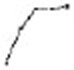	0.095
Severance	MCF	0.057	0.101	0.105	0.081	0.095	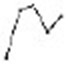	0.088
	DIVE	0.082	0.064	0.054	0.077	0.166	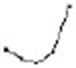	0.089
	MGLE	0.140	0.113	0.096	0.073	0.118	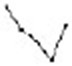	0.108
Efficiency	HULLA	0.382	0.388	0.367	0.365	0.350	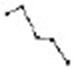	0.371
	HULLP	0.306	0.329	0.325	0.335	0.340	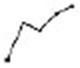	0.327
	HULLR	0.073	0.079	0.095	0.109	0.121	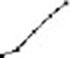	0.095
	HULLB	0.022	0.034	0.036	0.041	0.027	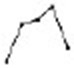	0.032
	HULLSI	0.256	0.326	0.311	0.298	0.280	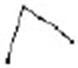	0.294

Connectivity is the second most important factor influencing the spatial heterogeneity of urban vitality. Well-connected street networks, which facilitate residents' physical activities in the neighborhood and enhance visual contact between people on the street, explain 44% of urban vitality. The mean q statistics of both connectivity in radius (CONN) and junctions in radius (JNC) reach 0.445 and 0.444, respectively. Approximately 44% of urban vitality is attributed to the number of street links and junctions. The explanatory power of these two variables on urban vitality has a slight upward trend and then decreases with the highest point at the spatial scale of 1,500 m.

Betweenness has maximum explanatory power for urban vitality of 46.7% at the spatial scale of 1,500 m. TPBTE, which reflects the quantity of visits per street link, is also non-negligible with the mean contribution of 30.1% to the pattern of urban vitality. Overall, the explanatory power of BTE demonstrates an inverted U shape over increasing spatial scales, whereas TPBTE reveals an N-shape pattern. TPD, which reflects the total flow to the destination, only explains 9.5% of urban vitality on average, with a slightly upward trend when spatial scale increases.

Among the metrics of closeness, NQPDE, which reflects network quantity and accessibility, contributes the most to the pattern of urban vitality, with the mean *q* statistic as high as 0.432. On average, ANGD explains 27.2% of urban vitality, indicating the importance of the angular analysis and cognitive difficulty during navigation. Mean Euclidean distance in radius (MED) explains the least variation in urban vitality with a mean *q* statistic of 0.109. A comparative analysis of these three variables illustrates that the conventional measure of street centrality by Euclidean distance is limited in its ability to reflect the subtleties in the network layout. The explanatory power of both NQPDE and ANGD increases as the distance interval increases.

Efficiency can explain 22% of the variation in urban vitality, and the communities with better spatial arrangements of street networks tend to be more vibrant. As a representative indicator reflecting network efficiency, HULLA can explain ~40% of urban vitality, although this figure decreases slightly over the incremental spatial scales. The explanatory power of HULLP demonstrates an upward trend from 30.6% at the spatial scale of 500 m to 34.0% at the spatial scale of 2,500 m. Convex hull shape index (HULLSI) contributes 29.4% to the heterogeneity of urban vitality, peaking at the spatial scale of 1,000 m. Therefore, the form of the overall spatial footprint of the network shapes the navigating efficiency of pedestrians and vehicles. The convex hull with a regular and straight-line shape can diversify the built environment and enhance urban vitality.

Severance, which reflects the navigating difficulties of pedestrians or vehicles, only explains 10% of the spatial heterogeneity of urban vitality. This illustrates the negative effect of network twistedness on creating a vibrant city. As spatial scale increases, the contribution of both DIVE and mean geodesic length in radius Euclidean (MGLE) demonstrates a U-shape pattern with the maximum value at the scale of 2,000 m. The explanatory power of severance metrics on urban vitality tends to increase rapidly because residents prefer simple routes when they choose to drive.

Therefore, their stratified spatial associations between various street network metrics and urban vitality are sensitive to spatial scales ranging from walking to driving distance. When people navigate street networks under different transport modes (e.g., walking, cycling, driving, etc.), the corresponding street network metrics within the walking or driving distance are different at multiple spatial scales. Thus, there is no single optimal scale for assessing urban vitality and designing street networks (50). Each scale from walking to driving distance enables different types of analysis and assessment about the vitality-led urban street design facilitating walking or driving. The spatial explicit analysis between the multi-scalar street network metrics and urban vitality yields important information for human-scale street design and land-use planning.

### Policy Implications

Interest in promoting a healthy, vibrant, and interactive neighborhood is a worldwide issue for various stakeholders in urban development. Good neighborhoods tend to have ideal environments that encourage walking, bicycling, and a sense of community, making them more spirited and livable (51). Examining the street configurations using sDNA reveals that conventional street centrality indices, such as accessibility and betweenness, cannot effectively guide the design of a good street network in real estate development. Two promising variables, namely, severance and efficiency, may provide some new design elements for streets. The geometric features of street networks, such as detours, shapes, and angular curvature, are important, as they influence people's subjective cognition when driving or walking. The irregular and complicated design of streets will multiply residents' navigating difficulties and threaten their psychological safety, causing people to stay indoors and making the community lifeless. Therefore, urban planners and real estate developers should design streets that benefit the physical and emotional health of children, seniors, and indeed every resident who plays a part in creating a truly safe and healthy neighborhood.

More strategies of vitality-related urban design should be encouraged to vitalize the traditional neighborhoods in the urban center and build new neighborhoods in the suburbs. These design elements are important to inform planners to create walkable, bike-friendly streets that are connected adequately to provide more walking routes. Streets that are less twisted and have regular shapes can make people feel psychologically safe, thus encouraging outdoor activities and enhancing personal interaction. At least one local main street with a straight-line shape in the community should be designed for pedestrians to meet, make friends, and share information, thus strengthening neighborhood bonds. Real estate developers should allocate space for recreational facilities and children's playgrounds on the local main street for residents to enjoy. Intersections should have regulations on driving speeds. Considering the aforementioned aspects, urban planners, real estate developers, policy makers, and non-profit representatives should devise appropriate street design guidelines for creating a healthy neighborhood.

Urban planners can better identify vitality by considering spatial dynamics in their assessments. In our study, notably, the downtown in the Chinese city of Wuhan is more vibrant than other areas. The street design in the downtown makes residents feel safe and comfortable while walking, creating a healthy, interactive neighborhood, while the less vital neighborhoods are mainly situated in the urban suburbs. The street design for the uptown area encourages people to drive, and blocks are often longer than 2,000 ft, which is less pedestrian-friendly. Street network design is an important way to strengthen urban vitality in the suburbs. A geospatial view can help urban planners and real estate developers target areas in which street design can be improved.

## Discussion

### Possible Mechanisms

It is interesting to explore the reason for the curious relation between geometry and urban vitality. Spatial network analysis provides extensive information about the metrics of street networks, as well as conventional accessibility and reachability. The correlation coefficients and the *q* statistics between various spatial network metrics and urban vitality from the multi-scalar perspective shed light on the details of their precise causal mechanisms.

Some metrics, such as density, connectivity, and betweenness calculated by spatial network analysis, are not new. These conventional street configurations provide an objective view of the location advantages of various places. Neighborhoods with densely distributed roads, high street connectivity, and many intermediary streets tend to attract more commercial and service activities, creating more employment. From a consumer's perspective, these network-based centrality metrics reflect how convenient access is to various services or facilities. From a vendor's perspective, central streets with high volumes of pedestrians or vehicles can provide larger market potential and more economic opportunities. Therefore, concentration, accessibility, livability, and diversity are closely linked with these centrality-based network measures.

Spatial network analysis provides a new view of the geometry of street networks, including detours, shape, and angular curvature, which are closely associated with people's subjective cognition. These geometric measures reflect the cognitive difficulties residents experience while walking or driving. Spatial network analysis includes a novel measure of closeness, ANGD, which calculates the distance in terms of angular changes, such as corners on links and turns at junctions. Residents who live in neighborhoods with high ANGD encounter more navigating difficulties en route to destinations, including traffic lights at crossroads and the need to make more turns. The severance and efficiency metrics are also new in spatial network analysis. When navigating a street network with high severance, the twisted streets make pedestrians or drivers feel psychologically insecure, decreasing the traffic flow and ultimately weakening urban vitality in the neighborhood. The efficiency metrics that consider the network shape directly represent the intrinsic navigability by foot. Efficient street networks are easily navigated by pedestrians, increasing residents' contact opportunities and making the local communities more active. The other possible causal mechanism of efficiency metrics on urban vitality is that high values of HULLA, HULLP, and HULLSI may indicate a long, straight pedestrian route in the local community. Such a road would be convenient in a small district and provide opportunities for people to interact with each other, ultimately making the community more lively.

The multi-scalar perspective is important for spatial network analysis to characterize the street configurations under different traveling scenarios. Defining a scale of interest is the key component of spatial network analysis. In the analysis of urban vitality, the scale tends to match different traveling scenarios from walking distances (up to 1,500 m or less) to driving distances. Table 3 shows that the metrics of density, connectivity, betweenness, and efficiency under the walking mode have more explanatory power over urban vitality. The densely distributed, well-connected, and efficient streets provide a walking-friendly environment for residents to strengthen communications and ultimately create a lively neighborhood. However, metrics such as angular curvature and severance are more important for understanding travel by car. People tend to drive when they must travel on streets with more angular changes and twistedness. In the Chinese context, the spatial design of street networks in downtown areas facilitates people's ability to walk around, which explains the high urban vitality.

### Strengths and Limitations

Prior studies have widely confirmed the associations between street centrality and land-use intensity, the location of economic activities, and social cohesion (52). However, spatial network analysis can better measure the details of network geometry, such as network shape, detours, and angular changes. These geometric details of street networks are related to the cognition difficulties experienced by pedestrians or drivers and whether they feel comfortable or safe psychologically. Thus, severance and efficiency are two promising parameters to provide a comprehensive view of the street network geometry. The other strength of spatial network analysis is the multi-scalar measure of street network characteristics, which allows the modeling of different navigating scenarios from walking to driving. The multi-scalar perspective allows urban planners and policy makers to design pedestrian streets or roads in ways that strengthen urban vitality.

One limitation of this study is its failure to consider transportation capacity and multiple transport modes like railways, subways, and highways. Although long, straight roads probably bring more traffic flows, the incorporation of traffic variables into spatial network analysis tends to enhance the relationship between street configurations and urban vitality. The other limitation is the lack of clarity of the causal mechanisms linking each street network metric to urban vitality. Although this study has provided insightful views about the possible causal mechanisms, a further detailed investigation is required to create vibrant neighborhoods by designing walkable and drivable streets.

## Conclusions

This study has explored the influence of spatial network layouts on urban vitality using geographic big data for Wuhan, an inland city in China. Concentration, accessibility, livability, and diversity are four major components that characterize urban vitality in Wuhan. The new technique of sDNA was employed to measure street configurations, including density, connectivity, closeness, betweenness, severance, and efficiency, from a multi-scalar perspective. Furthermore, the stratified spatial heterogeneity between street network metrics and urban vitality was investigated using the Geodetector tool. The following conclusions can be drawn.

First, the areas with the highest levels of urban vitality are clustered in the downtown area, whereas the uptown area is characterized by low urban vitality. Concentration, accessibility, and livability demonstrate a declining trend in concentric rings, whereas livability reveals a fluctuated upward trend. Second, 16 variables representing connectivity, closeness, betweenness, severance, and efficiency are computed. The correlation between these network characteristics and urban vitality is sensitive to different spatial scales. Third, the influence of street network layouts on urban vitality varies at multiple scales. Overall, connectivity has the largest explanatory power for urban vitality, amounting to over 40%, whereas betweenness and closeness have similar explanatory power of ~28%. Efficiency and severance contribute 22 and 10% to the spatial heterogeneity of urban vitality, respectively.

These conclusions shed light on the mechanisms between street configurations and urban vitality from the multi-scalar perspective. In the future, more strategies of vitality-based urban design should be encouraged to revitalize traditional downtown neighborhoods and build new neighborhoods in the uptown area. Multiple stakeholders, such as urban planners, real estate developers, policy makers, and non-profit representatives, should collaborate to devise effective street design guidelines for creating healthy, vibrant neighborhoods.

## Data Availability Statement

The raw data supporting the conclusions of this article will be made available by the authors, without undue reservation.

## Author Contributions

CF contributed to paper writing and spatial data analysis. SH contributed to data analysis and paper revision. LW contributed to result analysis and map designing. All authors contributed to the article and approved the submitted version.

## Conflict of Interest

The authors declare that the research was conducted in the absence of any commercial or financial relationships that could be construed as a potential conflict of interest.

## Footnotes

^1^In the era of globalization, the phenomenon of cities growing slowly or declining has become a significant international political and economic issue. A shrinking city can be characterized by population loss, financial crisis, employment decline, and other social problems.

^2^In China, the hierarchical administrative structure is provinces/prefecture-level cities/counties/towns. In each prefecture-level city, there are many city districts in charge of *jiedao*-level units. The community neighborhood is the lower governance unit of *jiedao*s.

^3^The popular interpolation methods include inverse distance weighted (IDW) and kriging. The authors use these two methods to calculate the pattern of housing prices and age. By comparing the model performances, the results produced by kriging conform to the spatial trend of housing prices and age with lower prediction errors over the map.
